# The Women in Medicine Summit (WiMS): Engaging students to identify and address gender-associated challenges in medicine

**DOI:** 10.36834/cmej.68714

**Published:** 2020-08-06

**Authors:** Flora Jung, Lily Wang, Jennifer Tang, Sophia Wen

**Affiliations:** 1 Faculty of Medicine, University of Toronto, Ontario, Canada

## Implication Statement

The Women in Medicine Summit (WiMS) is a novel, student-led conference initiated at the University of Toronto to engage the medical community in a discussion about unique gender-distinctive challenges in medicine. We identified topics inadequately addressed in undergraduate curriculums, including techniques to address microaggressions and balance work-life commitments. Students from five Canadian medical schools attended WiMS in 2018. Attendees perceived significant improvement in readiness in identifying and resolving gender-associated issues following the conference. Our findings may be used to motivate curriculum development and adoption of similar initiatives to improve education on diversity.

## Déclaration des répercussions

Le Sommet sur les femmes en médecine (WiMS) est une nouvelle conférence menée par des étudiants et lancée à l’Université de Toronto pour amener la communauté médicale à discuter des enjeux associés au genre en médecine. Nous avons relevé des sujets insuffisamment traités dans les programmes d’études de premier cycle, y compris des techniques pour examiner les micro-agressions et équilibrer les engagements professionnels et la vie privée. Des étudiants de cinq facultés de médecine canadiennes étaient présents au WiMS en 2018. Les participants ont perçu une importante amélioration dans leur capacité à discerner et à résoudre les enjeux associés au genre à la suite de la conférence. Nos résultats peuvent être utilisés pour inciter le développement de cursus et l’adoption d’initiatives similaires pour améliorer l’éducation sur la diversité.

While great strides have been made in achieving gender equity and diversity in medicine, female physicians remain disproportionately represented in leadership. In 2018, 2 of 17 (11.7%) Canadian Deans of Medicine and 6 of 26 (23%) CMA Board of Directors were women, despite 41% of Canadian physicians being women.^1^ 45-70% of American and Canadian medical school faculty reported curricular coverage of sex- and gender-based evidence as “minimal”.^2^^,^^3^ With recent increases in awareness, a parallel increase in efforts to fill this gap through curricular and non-curricular initiatives were seen.^4^^,^^5^

In 2018, University of Toronto students launched the Women in Medicine Summit (WiMS) to contribute to these efforts. WiMS aimed to assess gender-associated challenges faced by medical trainees and to evaluate WiMS’ impact on attendees. Conferences organized by groups like the Federation of Medical Women of Canada and Canadian Women in Medicine encourage student attendance. However, WiMS 2018 was the first to be organized by and for Canadian medical students with topics unique to the undergraduate experience. The local research ethics board deemed this program evaluation exempt from ethics review.

The full day summit featured two keynote speakers, three panels, and three workshops. 106 medical trainees from five Canadian medical schools attended the inaugural event on August 18, 2018. Topics addressed included leadership and power hierarchies, microaggressions, and the diversity of career paths. 55.8% of attendees who completed the post-program assessment (24/43, Appendix A) reported experiencing gender-associated discrimination in medicine, most frequently as microaggressions, where female medical trainees were mistaken as nurses or counseled against surgical specialties by faculty. When asked which gender-associated topics attendees felt were inadequately addressed by their curriculums, the following were thematically identified: recognizing gender-based and domestic violence as a health-care provider, identifying and addressing gender-based stigma in physician-patient relationships, and appraising research with poor gender representation. Attendees reported that WiMS significantly improved their readiness to address microaggressions, achieve work-life balance, and adopt different leadership techniques. 97.7% (42/43) of respondents reported learning something new at WiMS. 16.3% (7/43) and 72.1% (31/43) of Canadian medical students reported feeling “very” prepared to address gender-associated challenges in medicine before and after attending WiMS, respectively. All respondents reported WiMS would be valuable for other medical students.

The applicability of our results may be limited to the early experiences of medical school as 77% (33/43) of respondents were pre-clerks. We aim to improve attendance by clerks and allies in future annual iterations to elicit their important perspectives on workplace culture.

Our results identify an extra-curricular opportunity to help address gender in undergraduate medicine. Feedback from WiMS 2018 suggests that it was an effective short-term intervention to help Canadian medical trainees feel more prepared to manage gender-associated challenges in clinical settings. WiMS has continued to garner positive response, with the most recent WiMS 2019 hosting 10 of 13 Canadian medical schools and American delegates. Based on this interest and our results, we believe that other medical schools may benefit from adopting a similar initiative to improve education on gender-associated medical challenges, and by utilizing topics identified by our attendees to inform curriculum development.

## Appendix A: WiM Summit – feedback form

Please fill out the following form. Following completion of the anonymous survey, you will be taken to a separate form to enter the raffle.

Q1) What year of medical school are you in?

[ ] 1st [ ] 2nd [ ] 3rd [ ] 4th [ ] Graduated

Q2) What medical school did or do you attend?

Q3) Please indicate how prepared you felt to address gender-associated challenges in medicine prior this event.

1 2 3 4 5

Poor [ ] [ ] [ ] [ ] [ ] Excellent

Q4) Please indicate how prepared you felt to address gender-associated challenges in medicine NOW.

1 2 3 4 5

Poor [ ] [ ] [ ] [ ] [ ] Excellent

Q5) Have you personally faced any gender-associated discrimination or gender bias in medicine?

[ ] Yes

[ ] No

Q6) If comfortable, please share an example of a time when you experienced gender-associated bias or discrimination.

Q7) Consider health guidelines and research that reflect gender differences in medical care. How well are these covered by your school's curriculum?

1 2 3 4 5

Poor [ ] [ ] [ ] [ ] [ ] Excellent

Q8) If any, please indicate what gender-associated topics you believe could be covered more in undergraduate medicine.

Q9) Did you learn something new today?

[ ] Yes

[ ] No

Q10) If so, please share below.

Q11) Would events like this be valuable for other medical students to attend in the future?

[ ] Yes

[ ] No

Q12) Any other comments or suggestions for next year?
